# Supplementary material for: A Review of Digital Eye Strain: Binocular Vision Anomalies, Ocular Surface Changes, and the Need for Objective Assessment
Source: J Eye Mov Res. 2025 Sep 5;18(5):39. doi: 10.3390/jemr18050039 (PMC12452390; doi:10.3390/jemr18050039)
Supplement: Supplementary file 1 [file jemr-18-00039-s001.zip › jemr-3705160-supplementary.pdf]

Supplementary Materials: A Review of Digital Eye Strain: Binocular Vision Anomalies, Ocular Surface Changes, and the Need for Objective Assessment

[1] Ophthalmology Department, Unidade Local de Saúde de São José, 1150-199 Lisbon, Portugal; mjsbarata@gmail.com
[2] Institute for Research and Advanced Training, University of Evora, 7004-516 Evora, Portugal
[3] Department of Therapy and Rehabilitation Sciences, Escola Superior de Tecnologia da Saúde de Lisboa (ESTeSL), Instituto Politécnico de Lisboa, 1990-096 Lisbon, Portugal
[4] Comprehensive Health Research Centre (CHRC), Escola Nacional de Saúde Pública, Universidade NOVA de Lisboa, 1600-560 Lisbon, Portugal;
[5] Department of Health Strategies, National School of Public Health, NOVA University of Lisbon,
1600-560 Lisbon, Portugal; pedroaguiar@ensp.unl.pt
[6] Institute for Research in Ophthalmology, Foundation for Ophthalmology Development, 61-701 Poznan, Poland; ae.grzybowski@gmail.com
[7] Comprehensive Health Research Centre (CHRC), NOVA Medical School, Faculdade de Ciências Médicas, NMS, FCM, Universidade NOVA de Lisboa, 1169-056 Lisboa, Portugal
[8] NOVA Medical School, Faculdade de Ciências Médicas, NMS, FCM, Universidade NOVA de Lisboa,
1169-056 Lisboa, Portugal
[9] Division of Science, New York University Abu Dhabi, Saadiyat Marina District, Abu Dhabi P.O. Box 129188, United Arab Emirates
* Correspondence: andre.rosario@nms.unl.pt (A.M.-R.); carla.rita.costa@gmail.com (C.L.)
† These authors contributed equally to this work.

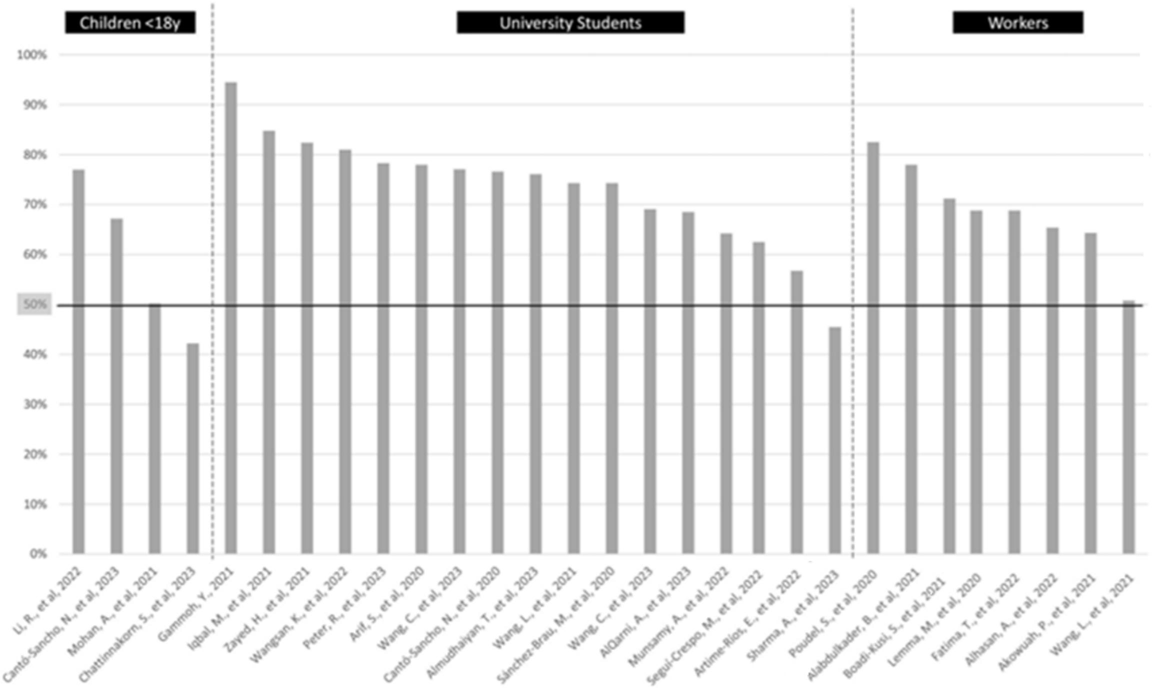

Children <18y
University Students
Workers
100%
90%
80%
70%
60%
50%
40%
30%
20%
10%
0%
Li, R., et al, 2022
Cantó-Sancho, N., et al, 2023
Mohan, A., et al, 2021
Chattinnakorn, S., et al, 2023
Gammoh, Y., 2021
Iqbal, M., et al, 2021
Zayed, H., et al, 2021
Wangsan, K., et al, 2022
Peter, R., et al, 2023
Arif, S., et al, 2020
Wang, C., et al, 2023
Cantó-Sancho, N., et al, 2020
Almudhaiyan, T., et al, 2023
Wang, L., et al, 2021
Sánchez-Brau, M., et al, 2020
Wang, C., et al, 2023
AlQarni, A., et al, 2023
Munsamy, A., et al, 2022
Seguí-Crespo, M., et al, 2022
Artime-Ríos, E., et al, 2022
Sharma, A., et al, 2023
Poudel, S., et al, 2020
Alabdulkader, B., et al, 2021
Boadi-Kusi, S., et al, 2021
Lemma, M., et al, 2020
Fatima, T., et al, 2022
Alhasan, A., et al, 2022
Akowuah, P., et al, 2021
Wang, L., et al, 2021

The bar chart presents the prevalence of DESS across various age groups and occupational categories.

Figure S1. Prevalence of Digital Eye Strain Syndrome as reported in 29 English-language manuscripts published between 2020 and 2024.

Table S1a Newcastle-Ottawa quality assessment scale for the cohort studies included in the review.

	**Study**											
	**Golebiowski, B., et al., 2020 [15]**	**Sánchez-Brau, M., et al., 2020 [33]**	**Yammouni, R., & Evans, B., 2020 [19]**	**Sanchez-Valerio, M., et al., 2020 [16]**	**De-Hita-Cantalejo, C., et al, 2021 [34]**	**Liu, Z., et al., 2022 [35]**	**Auffret, E., et al., 2022 [36]**	**Wang, J. et al., 2022 [37]**	**Maharjan, U., et al., 2022 [21]**	**Agarwal, R., et al., 2022 [4]**	**Patel, H., et al., 2023 [42]**	**Cacho-Martínez, P., et al., 2024 [32]**
**Selection**												
Representativeness of the exposed cohort		*										*
Selection of the non exposed cohort	*	*	*	*	*	*	*	*	*	*	*	*
Ascertainment of exposure	*	*	*	*	*	*	*	*	*		*	*
Demonstration that outcome of interest was not present at start of study												
**Comparability**												
Comparability of cohorts on the basis of the design or analysis	*	*	*	*	*	*	*	*	*	*	*	*
**Outcome**												
Assessment of outcome	*	*	*	*	*	*	*	*	*		*	*
Was follow-up long enough for outcomes to occur	*	*	*	*	*	*	*	*	*		*	
Adequacy of follow up of cohorts							*					
**Total score**	5	6	5	5	5	5	6	5	5	2	5	5

Table S1. b. Newcastle-Ottawa quality assessment scale for the Case-Control study included in the review.

	**Study**
	De-Hita-Cantalejo, C., et al, 2022 [38]

Selection	
Is the case definition adequate?	*
Representativeness of the cases	*
Selection of Controls	*
Definition of Controls	*
**Comparability**	
Comparability of cases and controls on the basis of the design or analysis	*
**Outcome**	
Ascertainment of exposure	*
Same method of ascertainment for cases and controls	*
Non-Response rate	
**Total score**	7

Table S2. Cochrane risk-of-bias tool for randomized trials (RoB 2) for studies included in the review.

**Study: Yuan K, Zhu H, Mou Y, Wu Y, He J, Huang X, et al. Effects on the Ocular Surface from Reading on Different Smartphone Screens: A Prospective Randomized Controlled Study. Clin Transl Sci. 2021;14(3):829–36. [17]**	
**Risk of bias assessment – Domain**	**Risk-of-bias judgement**
Bias arising from the randomization process	Some concerns<br>Although the study reports that participants were randomised into four groups, it fails to describe the method used for randomisation
Bias due to deviations from intended interventions	Some concerns<br>Participants were aware of the screen type (OLED vs eINK), as these technologies are visually distinct. However, the environment was standardised, and reading time was controlled.
Bias due to missing outcome data	Low risk of bias<br>No missing. All 119 participants completed the assessments and were included in the analyse.
Bias in measurement of the outcome	Some concerns<br>Whilst numerous outcomes were objective in nature (NIBUT, FBUT, CFS), the subjective symptom assessments (OSDI, CVS-Q) might have been subject to bias due to the absence of participant blinding.
Bias in selection of the reported result	Some concerns<br>The outcome measurement method was appropriate and likely consistent across intervention groups. However, it is unclear whether outcome assessors were blinded, which could have introduced bias.
Overall risk of bias	Some concerns<br>Although the study is operationally well conducted, the lack of blinding and insufficient description of the randomisation process warrant caution.
**Study: Mou Y, Shen X, Yuan K, Wang X, Fan F, Wu Y, et al. Comparison of the influence of light between circularly polarized and linearly polarized smartphones on dry eye symptoms and asthenopia. Clin Transl Sci. 2022;15(4):994–1002.**	
**Risk of bias assessment – Domain**	**Risk-of-bias judgement**
Bias arising from the randomization process	Low risk of bias<br>Participants were randomly assigned to four equally sized groups, using a statistical formula for randomisation.

Bias due to deviations from intended interventions	Low risk of bias<br>This was a double-blind study in which both participants and examiners were unaware of group allocation, as the smartphones used were visually identical. The protocol was strictly adhered to.
Bias due to missing outcome data	Low risk of bias<br>No missing. All 120 participants completed the assessments and were included in the analyse.
Bias in measurement of the outcome	Low risk of bias<br>Objective measurements were conducted using standardised instruments, with assessors blinded to group allocation. The subjective scales employed were validated.
Bias in selection of the reported result	Low risk of bias<br>The outcome measurement method was appropriate, with no apparent differences between intervention groups. Outcome assessors were blinded, and the evaluation did not seem to be influenced by knowledge of the intervention received.
Overall risk of bias	Low risk of bias<br>The study was well conducted, with appropriate blinding, clear randomisation, no missing data, and standardised assessment tools. The findings were fully reported and aligned with the study objectives.
